# Higher serum uric acid is associated with poorer cognitive performance in healthy middle-aged people: a cross-sectional study

**DOI:** 10.1007/s11739-023-03337-1

**Published:** 2023-06-17

**Authors:** Yousef Khaled, Aya A. Abdelhamid, Hissa Al-Mazroey, Abdulrahman K. Almannai, Sara Fetais, Aisha S. Al-Srami, Shaima Ahmed, Noora Al-Hajri, Ayman Mustafa, Tawanda Chivese, Laiche Djouhri

**Affiliations:** 1 Department of Basic Medical Sciences, College of Medicine, QU Health, Qatar University, P.O. Box 2713, Doha, Qatar; 2 Biomedical and Pharmaceutical Research Unit, QU Health, Qatar University, Doha, Qatar

**Keywords:** Cognitive function, Uric acid, Qatar Biobank (QBB)

## Abstract

Age-related cognitive impairment can occur many years before the onset of the clinical symptoms of dementia. Uric acid (UA), a metabolite of purine-rich foods, has been shown to be positively associated with improved cognitive function, but such association remains controversial. Moreover, most of the previous studies investigating the association included elderly participants with memory-related diseases. Therefore, the present study aimed at investigating whether serum UA (sUA) is associated with cognitive performance in healthy middle-aged individuals. We conducted a cross-sectional study on a cohort of middle-aged individuals (40–60 years old) who participated in the Qatar Biobank. The participants had no memory-related diseases, schizophrenia, stroke, or brain damage. They were divided according to sUA level into a normal group (< 360 μmol/L) and a high group (≥ 360 μmol/L), and underwent an assessment of cognitive function using the Cambridge Neuropsychological Test Automated Battery. Two cognitive function domains were assessed: (a) speed of reaction/reaction time and (b) short-term visual memory. The median age of the 931 participants included in the study was 48.0 years (IQR: 44.0, 53.0), of which 47.6% were male. Adjusted multivariable linear regression analyses showed that higher sUA is associated with poorer performance on the visual memory domain of cognitive function (β = − 6.87, 95% CI − 11.65 to − 2.10, *P* = 0.005), but not on the speed of reaction domain (β = − 55.16, 95% CI − 190.63 to 80.30, *P* = 0.424). Our findings support previous studies suggesting an inverse association between high sUA levels and cognitive function in elderly and extend the evidence for such a role to middle-aged participants. Further prospective studies are warranted to investigate the relationship between UA and cognition.

**Supplementary Information:**

The online version contains supplementary material available at 10.1007/s11739-023-03337-1.

## Introduction

Uric acid (UA) is a natural waste product from the digestion of purine-rich foods that are generally abundant in high-protein diets [1]. It is formed in the body (mainly in the liver) from the exogenous purines and endogenously from damaged, dying and dead cells [2]. About 90% of the filtered UA in kidney is reabsorbed by the renal tubules, and only about 10% is excreted in the urine as a way of disposing of unwanted amino acids and nitrogen (see [2]) for review). The serum UA (sUA) level is the result of a balance between dietary intake of purines activity of xanthine oxidase, and renal UA excretion [3]. UA is a very potent free radical scavenger and is, therefore, considered one of the most important antioxidants in human plasma [4]. On the other hand, UA can become a pro-oxidant by forming radicals in reactions with various other oxidants causing inflammation (see [2]). Thus, UA seems to play a dual role as a pro- and antioxidant (see [2]), and the balance between the two effects seems to depend on a complex interplay between various factors including UA concentration, the nature and concentration of free radicals, and other antioxidant molecules (see [2]). It is worth mentioning, in this context, that the brain has a lower antioxidant capacity than other organs, which makes it particularly vulnerable to oxidative stress (see [5]), and that oxidative damage in the CNS results from oxidation and nitration of proteins, lipids, and DNA, leading to necrosis and apoptosis of neuronal cells (see [2]).

Cognition consists of major cognitive domains including learning, memory, language, attention and executive and visuospatial functions (see [2]). These domains can be affected/impaired by various processes, toxins and age-related neurocognitive disorders including those that do not cause sufficient impairment to qualify for a diagnosis of dementia [6]. Numerous studies have investigated the physio-pathological role of UA in cognitive function, but yielded conflicting results. Indeed, several studies reported that UA has a protective role against the progression of cognitive impairment, and that higher sUA level is associated with better cognitive performance, owing to the antioxidant property of UA [7–13]. In contrast, other studies showed that elevated sUA is associated with poorer cognitive function owing to the pro-oxidant character of sUA [14–17]. Taken together, the findings of these various studies indicate that the association between sUA level and cognitive function remains controversial.

Conflicting findings have also been reported in studies that investigated the effects of UA on cognitive function in individuals with different types of dementia (see Discussion). Seemingly, however, only two previous studies investigated the relationship between sUA and cognitive functioning in apparently healthy participants. One of those studied was a cross-sectional study on a very small sample (*n* = 96) of elderly Americans [18]. The findings of this study showed that participants with mildly elevated sUA were more likely to score in the lowest quartile of the sample on measures of cognitive functions including processing speed and verbal and working memory. In contrast to this finding, sUA was associated with better performance on an executive function test in men, but not in women in a larger middle-aged subset population (*n* = 12,215; < 65 years old) from the ELSA-Brasil cohort study[19].

It is noteworthy that neuropathological lesions can appear 20 years before the onset of the clinical symptoms of dementia [20]. Given the long-preclinical phase of dementia, studies on younger participants would be valuable for understanding potential associations between risk factors and cognition at the preclinical stages of dementia. Therefore, the aim of this study was to examine whether there is an association between sUA levels and cognitive performance in healthy middle-aged (40–60 years old) participants.

## Materials and methods

### Study design

We conducted a cross-sectional study on participants who were randomly chosen from the Qatar Biobank (QBB). The data from these participants were collected during the period of 2014 to 2020. The QBB is a national resource that collects samples and information on different aspects of health and lifestyle of volunteers both citizens and long-term residents in Qatar to participate in a longitudinal observational cohort [21]. The design and methods of the QBB have been described elsewhere [21]. Participants who had UA measurement and cognitive assessment were randomly selected and included in the present study. Ethical approval for this study was provided by the QBB (Ref—EX-2019-RES-ACC-0182-0107). During their participation in the QBB, participants gave written informed consent. The research also received waiver of ethics approval from the Qatar University Institutional Review Board (Ref—QU-IRB 1223-E/20).

### Study population

**Fig. 1:**
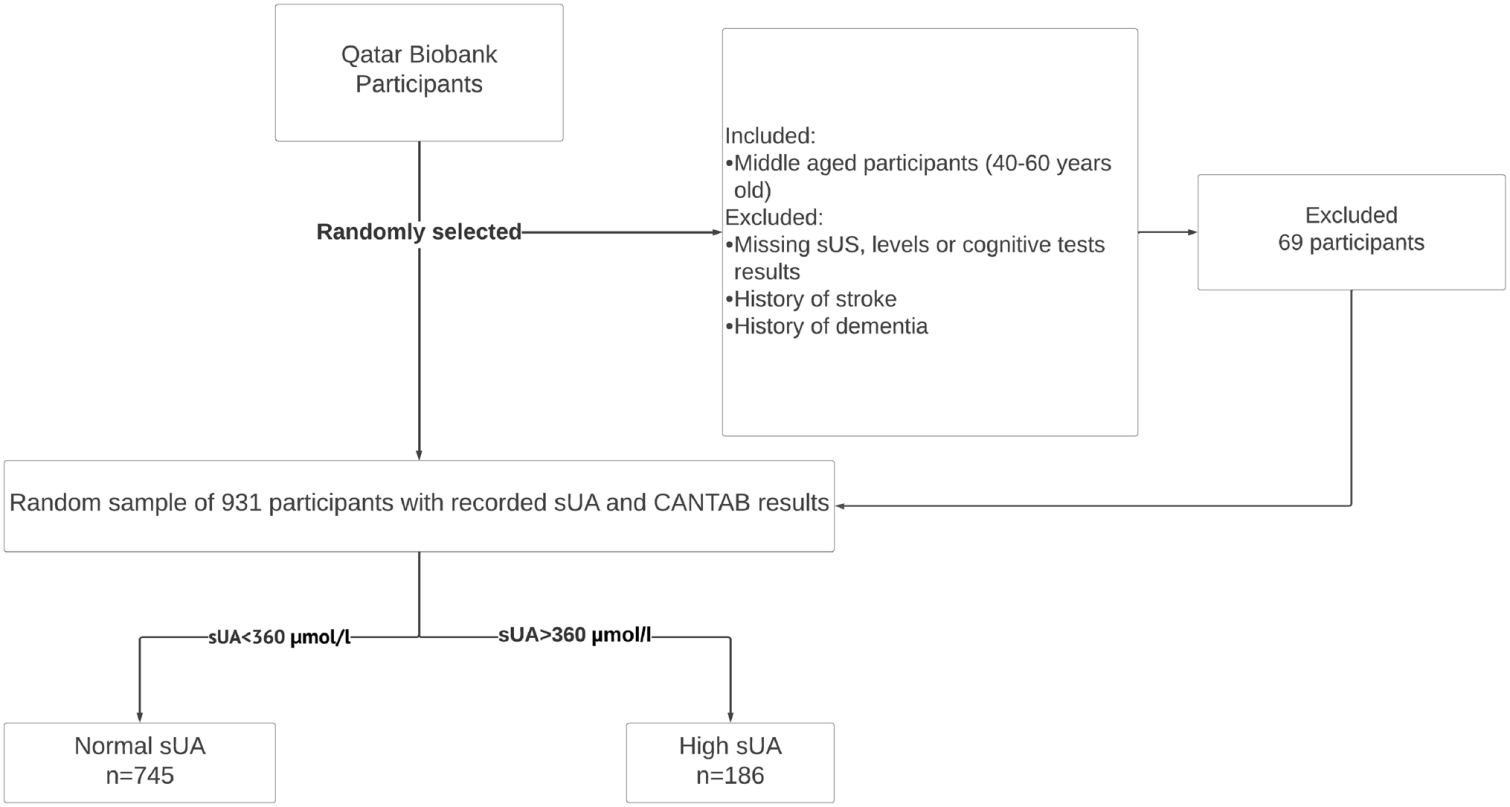
Flow diagram depicting the procedure for selecting eligible middle-aged participants (*n* = 931)

Participants included in the present study were adults aged 40–60 years. We excluded participants with memory-related diseases (e.g., Alzheimer’s disease or any type of dementia), history of mental disorders such as schizophrenia, epilepsy or brain damage. Participants who did not have either sUA levels measured or did not perform the cognitive test were also excluded. Further, given that stroke can cause development of dementia or cognitive impairment [21] and is associated with high serum UA levels [22], any participants with history of stroke were also excluded (Fig. 1).

### Data

The data were provided by QBB, and included personal medical history, laboratory results including sUA levels and comorbidities. The data also included the results of cognitive function tests conducted using the Cambridge Neuropsychological Test Automated Battery (CANTAB) screening tool (see below). In addition, the following data were obtained for each participant through a self-reported questioner that included detailed questions about health conditions, smoking habits and socio-demographic factors: demographic data including age, gender, education level, marital status, nationality, occupation, and family medical history. Data were also obtained on chronic disease history, blood pressure and anthropometric measurements (BMI, waist-to-hip ratio, systolic blood pressure, diastolic blood pressure, mean arterial pressure, and use of antihypertensive medication), history of cardiovascular diseases (confirmed diagnosis of stroke and acute coronary syndrome). sUA levels were measured in the laboratories of Hamad Medical Centre Laboratory in Doha using the Enzymatic colorimetric test (Uricase).

### Assessment of cognitive function

Each participant underwent an assessment of cognitive function using the Cambridge Neuropsychological Test Automated Battery (CANTAB) [23]. The CANTAB battery focuses on several cognitive domains including working memory and planning, attention, and visuospatial memory. Unfortunately, however, we had access to the data of only two tests: (a) Paired Associate Learning (PAL) test that assesses visual memory and new learning, and (b) the Reaction Time (RT) test that assesses speed of response and movement [15].

#### Paired associate learning (PAL) test

As mentioned above, this test (also known as the paired episodic memory test) assesses visual memory and new learning. It evaluates the short-term memory function of participants. In this test, the participants learn the position of an increasing number of cards. The test uses a paired associate learning task. In this task, a series of cards with different patterns are opened randomly and need to be learned within a short time period. Then, the cards are closed, and the patterns are displayed one at a time in the middle of a screen. The participant must select the card that originally contains the pattern. If the participant failed to choose the correct card, the position of each card is presented for 2 s consecutively, but not sequentially. The participant has to recall the location of each card. The test has seven levels, with the first level having two cards and the seventh level having eight cards. Participants proceed to the next level only after successfully completing the previous level. The number of attempts needed to identify the location of each card correctly is summed to achieve a total guess. The response duration is the total time the participant takes from the moment the pattern is presented in the middle of screen until he/she selects a card. The outcomes for each participant were the number of levels reached, the total guesses and the time taken. The PAL test evaluates both frontal and the temporal lobe functions, but is particularly dependent on integrity of the entorhinal and hippocampal areas of the brain (see [24] and references therein).

#### Reaction time (RT) test

This test assesses motor and mental response speeds, as well as movement time, reaction time, response accuracy, and impulsivity [25]. It is designed to measure the subject’s speed of response to a visual target, which we refer to throughout the paper as the processing speed. It is divided into five stages, each successive stage having increasingly complex response requirements [23]. The test comprises of 60 trials per participant. The task generates 60 presentations of one of two targets. The target is presented as a small white box within one of two larger black boxes. The location of the target within the black box varies. During each trial, the participant has to select the box on the screen where the target appears as quickly as possible. The outcomes were the product of the number of mistakes made while performing the test multiplied by the time taken during all 60 trials. The generated score reflects both variables collectively, and therefore, the participant with the lowest score had the best performance.

### Data analysis

Participants were categorized into high and normal UA groups, according to their sUA levels, using a cutoff of 360 μmol/L (< 6.0 mg/dL) used as the sUA goal in gout management as reported previously [26]. This is because there is evidence suggesting an adverse role of uric acid, below the current cutoff for hyperuricemia (416 μmol/L), in musculoskeletal damage, traditional cardiovascular disease risk factors such as hypertension and diabetes, cardiovascular disease, and renal disease (see [26]). The participants’ characteristics were described for the entire sample and for the two groups defined according to sUA levels. The descriptive data were first explored for normality using histogram and the Shapiro–Wilk test. Continuous variables were described as means and standard deviations (SD) or as median and quartiles if not normality distributed. Categorical variables were compared using the Chi-squared test. Numerical data were compared either using the t-test or the Mann–Whitney *U* test, if not normally distributed.

Best performance in the PAL test is when completing all seven levels with least sum of total guesses and least time, and vice versa for the worst performance. Participants were ranked from best performance to the worst performance. The memory performance score was assigned in such a way that the worst performer had a score of zero and the best performer had a score of 100. For the reaction time test, a score is generated by the product of the total mistakes a participant made in the 60 trials multiplied by the time they took. The generated score reflects both variables collectively, and therefore, the participant with the lowest score had the best performance.

We compared cognitive function across the high and normal UA groups using two multivariable linear regressions, one for each test. In the first regression, the performance variable of the PAL test was the dependent variable and categorized UA was the independent variable. In the second regression, the score (product of sum of response and sum of mistakes) of the reaction time test was the dependent variable and categorized UA was the independent variable. Based on evidence from previous studies we adjusted, in both regressions, for age [27, 28], BMI [1, 29], gender, education [30, 31], hypertension and diabetes.

## Results

### Characteristics of participants

A total of 931 participants were included in the present study. Most (88.5%) were of Qatari nationality. The clinical and demographic characteristics of the participants are shown in Table 1. Their median age is 48.0 years (IQR 44.0–53.0) with no difference between individuals with high and those with normal sUA. 24.6% of the participants had diabetes while 10.7% had hypertension. As already mentioned, the division of participants into high (≥ 360 μmol/L) and normal (< 360 μmol/L) groups was based on a cutoff of 360 μmol/L reported previously [26] sUA levels ranged from 171 to 585 µmol/L in men and 97 to 552 µmol/L in women. Of the 931 participants, 186 individuals had high sUA and 745 had normal sUA (Table 1 and Fig. 1). Most (85.5%) of those with high sUA were males (Table 1). As expected, the mean sUA level in men (337.9 ± 3.2) was significantly (*P* < 0.0001) higher than that in women (258.7 ± 2.8). The characteristics of participants were presented for females and males separately in Supplementary Tables 1 and 2, respectively.

**Table 1:** Main characteristics of the study sample according to the serum uric acid levels (*n* = 931)

Factor	Level	Total	Normal uric acid	High uric acid	*p*-value
N		931	745	186	
Age, median (IQR)		48.0 (44.0, 53.0)	48.0 (44.0, 53.0)	47.0 (44.0, 52.0)	0.048
Age categories, n (%)	40–49 years	560 (60.2%)	434 (58.3%)	126 (67.7%)	0.018
	50–60 years	371 (39.8%)	311 (41.7%)	60 (32.3%)	
Nationality, n (%)	Non-Qatari	107 (11.5%)	85 (11.4%)	22 (11.8%)	0.87
	Qatari	824 (88.5%)	660 (88.6%)	164 (88.2%)	
Gender, n (%)	Female	488 (52.4%)	463 (62.1%)	25 (13.4%)	< 0.001
	Male	443 (47.6%)	282 (37.9%)	161 (86.6%)	
Education, n (%)	Primary or below	70 (7.5%)	65 (8.7%)	5 (2.7%)	0.008
	Secondary	262 (28.2%)	214 (28.8%)	48 (25.8%)	
	Tertiary	598 (64.3%)	465 (62.5%)	133 (71.5%)	
BMI, median (IQR)		29.2 (26.3, 32.9)	29.1 (26.1, 32.8)	29.6 (27.1, 33.1)	0.073
Cigarette smoking, *n* (%), (*n* = 618) *	Non-Smoker	451 (77.8%)	335 (79.8%)	116 (72.5%)	0.16
	Current Smoker	125 (21.6%)	82 (19.5%)	43 (26.9%)	
Shisha, *n* (%), (*n* = 618)	Non-Smoker	4 (0.7%)	343 (80.9%)	115 (72.8%)	0.034
	Current Smoker	458 (78.7%)	81 (19.1%)	43 (27.2%)	
Diabetes, *n* (%)		229 (24.6%)	193 (25.9%)	36 (19.4%)	0.063
Hypertension, *n*		100 (10.7%)	73 (9.8%)	27 (14.5%)	0.063
Memory test performance, mean (SD)		51.3 (28.6)	51.7 (29.0)	50.0 (27.0)	0.47
Memory test performance, median (IQR)		52.1 (26.9, 76.2)	53.0 (25.6, 77.1)	48.0 (30.6, 71.8)	0.45
Reaction test (total time × total mistakes), mean (SD)		287.1 (779.5)	315.0 (855.6)	175.6 (308.4)	0.029
Reaction test (total time × total mistakes), median (IQR)		95.3 (52.1, 203.3)	96.4 (53.7, 222.2)	86.8 (50.3, 157.3)	0.032

Continuous variables represented as mean ± SD and median (IQR), interquartile range), categorical variables as *n* (%)

*BMI* body mass index, *IQR* interquartile range. Memory performance score ranged from 0 (the worst) to 100 (the best). Reaction time score was calculated as total time taken multiplied by the total mistakes. A higher product of time and mistakes indicated worse performance and vice versa

### Comparison of cognitive function by uric acid level

**Fig. 2:**
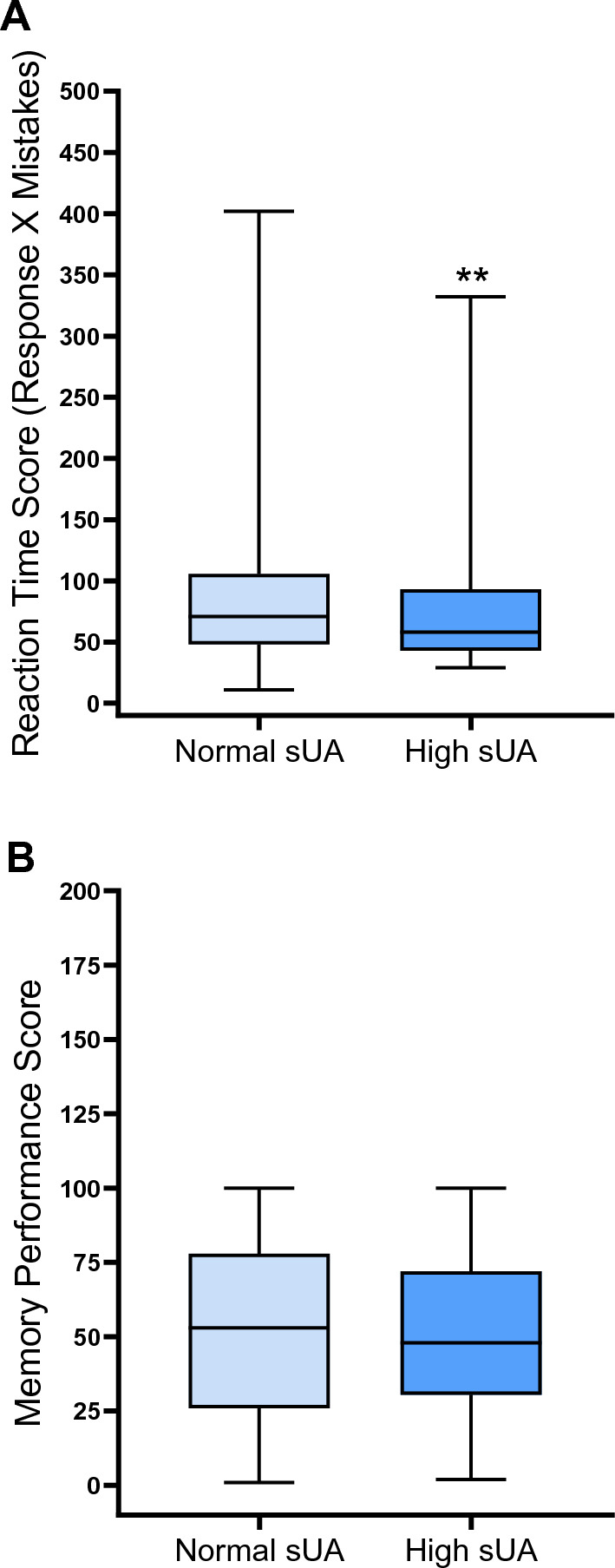
Box plots comparing performance of patients with high sUA and normal sUA in the reaction time test (**A**) and the memory test (**B**). Participants with high sUA had a significantly (*P* < 0.01) lower median score in the reaction time test compared with the normal sUA group (**A**). Note that low scores indicate better performance in this test. However, in the PAL test (**B**), there was no significant difference in the median performance score between participants with high sUA and those with normal sUA. Memory performance score ranged from 0 (the worst) to 100 (the best). Comparison was made using Mann–Whitney *U* test. **P < 0.01

To examine the relationship between cognitive performance and sUA, we compared the performance of the high sUA group with that of the low sUA group on the reaction time and the PAL tests (Table 1, and Fig. 2). As shown in Fig. 2A, participants with high sUA had a significantly (*P* < 0.01) lower median score (86.8, IQR: 50.3, 157.3) in the reaction time test compared with the normal sUA group (96.4, IQR: 53.7, 222.2). It should be noted that a lower score in this test indicates better performance. However, in the PAL (paired episodic memory) test, there was no significant difference (*P* = 0.45) in the median performance score between participants with high sUA (48.0, IQR: 30.6, 71.8) and those with normal sUA (53.0, IQR: 25.6, 77.1), as shown in Fig. 2B.

### Association between high uric acid level and episodic memory and reaction time—multivariable linear regression

A multivariable linear regression analysis was conducted to determine the association between sUA and cognitive function in our cohort of 931 middle-aged participants. After adjusting for age, gender, BMI, education, hypertension and diabetes (Table 2), multivariable linear regression analysis showed that higher sUA was associated with poorer performance on the visual memory domain of cognitive function assessed with the PAL test (β = − 6.31, 95% CI − 11.17 to − 1.45 *P* = 0.011). However, there was no association between sUA and the processing speed domain assessed with the reaction time test (β = − 73.50, 95% CI − 208.81 to 61.81, *P* = 0.287). These findings suggest an inverse association between high sUA and visual memory and new learning domains of cognitive function but not on the processing speed function.

**Table 2:** Results of multivariable linear regression analyses showing that higher sUA is associated with poorer performance on the visual memory domain of cognitive function but not on the speed of reaction domain in middle-aged participants (*n* = 931)

	Coefficient	*P*-value	95% Confidence interval
Memory test performance			
Uric acid categories			
High UA	− 6.3	0	− 11.17 to − 1.45
Constant	38	< 0.001	25.13 to 51.67
Reaction test score			
Uric acid categories			
High UA	− 74	0.3	− 208.81 to 61.81
Constant	729	< 0.001	359.83–1098.10

Adjusted for age, gender, *BMI* education, diabetes and hypertension

To determine whether there was a sex difference in the association between sUA and cognitive function, the analysis was stratified by gender. The analysis showed that, in women, high sUA was associated with a higher decline in memory performance (β = − 10.78, 95% CI − 20.6 to − 0.91, *P* = 0.03) while a weak association was observed with processing speed (β = − 245.5, 95% CI − 570.77–79.75, *P* = 0.14), with the *p*-value suggesting weak evidence against the null hypothesis. In men, high sUA was associated with a significant decline in memory performance similar to the one seen in the whole study group (β = − 6.33, 95% CI − 11.69 to − 0.96, *P* = 0.02) but not with the processing speed (β = 15.45, 95% CI − 106.44 to − 137.34, *P* = 0.478). To explore these findings further, we divided the uric acid into tertiles. However, there were no significant associations between the different tertiles and cognitive function domains (Supplementary Table 3).

## Discussion

In the present study, we found in a cohort of apparently healthy middle-aged participants (aged 40–60 years old) without existing neurocognitive diseases, that higher levels of sUA were associated with poorer visual episodic memory, but not with the processing speed, assessed with the PAL test and the reaction time test respectively. This finding might be related to the findings showing that episodic memory is the earliest and most prominent change in patients with Alzheimer’s disease, and that the decline in the processing speed progresses linearly with age (see [24, 25]). It is noteworthy that UA could pass through the blood–brain barrier and be deposited at significant levels in the hippocampus [32], and that the hippocampus and associated areas are the critical brain regions for learning and memory. Indeed, several animal and human studies have shown that damage to the hippocampal formation results in substantial impairments in memory performance (see [24]). Thus, it is possible that the detrimental effects of UA on cognitive performance reported here are due to its pro-oxidant effects on the entorhinal and hippocampal areas because performance on the PAL test that we used to assess the visual episodic memory, is particularly dependent on the integrity of these cortical areas (see [24] and references therein).

Our findings of inverse relationship between sUA levels and cognitive performance are consistent with those of several previous studies conducted on elderly aged > 60 years in different regions [9, 10, 14, 15, 18]. Indeed, in a cohort study on 423 healthy community-dwelling old women (aged 70–79 years) residing in eastern Baltimore (USA) from the Women’s Health and Aging Study (WHAS II), higher sUA was found to be associated with poorer working memory after adjusting for several potential demographic and health confounders [14]. Similarly, in their Brisighella study (Italy), Cicero et al., [15] found that higher sUA levels were associated with cognitive impairment in a case–control study of pharmacologically untreated elderly subjects (*n* = 288, mean age 69 ± 6 years old). An earlier cross-sectional study on a very small sample of elderly Americans aged ≥ 65 (*n* = 96) also reported that even mild elevations of UA might increase the risk of cognitive decline [18]. Moreover, a recent study on older healthy Americans aged ≥ 60 years (*n* = 2767) reported that age had an impact on the association between sUA and cognition [33]. These co-workers found that older adults aged 60–69 exhibited a negative correlation, while those 70 and older showed a positive correlation. Our findings are also in line with those of Latourte et al. [16] who reported that high sUA increased the risk of dementia after following a cohort of 1598 heathy older people (72.4 ± 4.1 years: mean ± SD) for 12 years [16]. Thus, our findings provide further support to these studies showing a negative correlation between sUA and cognition in elderly (> 60 years old) and extend the current evidence for such association to younger population (≤ 60 years old).

In contrast to our findings, Baena et al. [19] found, in a large middle-aged subpopulation aged < 65 years from the ELSA-Brasil cohort study on 6751 women and 5464 men, that sUA was associated with better performance on an executive function test in men, but not in women. The apparent discrepancy between our and their findings might be due to the use of different screening tests, their older sample (participants > 60 years), or other factors such as health and nutritional state of the participants. Several other studies also suggested that high sUA levels are advantageous for general cognition including memory, language, processing speed, and attention, and that sUA is an independent risk factor for dementia ([3, 7, 22, 34, 35]. For example, a prospective cohort study on participants aged ≥ 55 years (*n* = 4618) showed that elevated sUA predicted a decreased risk of dementia after adjusting for several cardiovascular risk factors [7]. These co-workers also reported that, in a subsample of 1724 participants who remained free of dementia during follow-up, higher sUA at baseline predicted better cognitive performance later in life in the absence of cardiovascular risk factors [7]. sUA levels were also found to be lower in patients with Alzheimer’s disease and in individuals with mild cognitive impairment than those in healthy controls, suggesting that UA may have a protective effect [19]. Furthermore, the findings of both an Italian multi-center clinical trial on 232 participants [34] and a study on a total of 430 non-demented South Korean individuals aged between 55 and 90 [22], showed a decrease in serum sUA in patients with Alzheimer’s disease. In support of these findings, a meta-analysis of 53 studies showed that participants with dementia had lower sUA levels than those without dementia [35].

It is not clear why research on the association between sUA and cognitive dysfunction has yielded conflicting findings. However, some of the discrepancies between the various studies might be due to the differences in the type of cognitive tests used and/or other factors such as the sample size, the study methods, the age and characteristics of the participants, and/or undiagnosed conditions. For example, undiagnosed depression, which is prevalent among elderly populations, can affect cognition. Indeed, the memory complaints of 5% of referrals in the Cambridge Memory Clinic were attributed to depression [36]. Another important factor that is likely to account for the discrepancy between the studies is the health sate of the participants, and whether or not they have concurrent medical problems such as, malnutrition, diabetes, hypertension, renal failure, all of which have the potential to alter sUA and affect cognitive function. Participants with different subtypes of dementia may also exhibit varying relations between sUA and cognition because a meta-analysis [35] has shown UA to be a potential risk factor for dementia associated with Alzheimer’s disease and Parkinson’s disease, but not with vascular dementia. The diagnosis of dementia itself might also account for the differences between the various studies because in some studies only a provisional diagnosis is made of dementia or cognitive decline.

The precise mechanisms by which UA could influence cognitive function remain unknown. However, several factors are believed to be involved in the pathogenesis of cognitive impairment including oxidative stress that might result from chronic exposure to environmental toxins, low antioxidant levels in the brain free radical scavenging enzymes, or mitochondrial dysfunction [3]. sUA levels were found to be associated with UA from cerebrospinal fluid, which suggests an influence of UA on brain and cognitive system [36]. Thus, the adverse effects of UA on cognition may be due its pro-oxidant effects (see Introduction). Indeed, in the presence of pre-existing neuroinflammation, sUA can be oxidized by peroxidase to produce the potent oxidant urate hydrogen peroxide (see [2]). In addition, catabolism of xanthine by which UA is generated produces superoxide anions. These two effects can increase the oxidative stress that would result in cell damage and apoptosis [37]. In addition, sUA was found to be negatively correlated with superoxide dismutase level, indicating the effect of UA in increasing the oxidative stress [37]. These co-workers also found a positive correlation between sUA and the β-Amyloid peptide level, the main component of the amyloid plaque, a well-established factor of Alzheimer’s disease pathogenesis, further supporting the harmful effect of UA on cognition [37]. However, another study [22] did not find any associations of serum UA with β-Amyloid or tau pathologies. On the other hand, the beneficial effects on cognition reported by other studies might be due its antioxidant characteristics (see [2]).

Our current study has several strengths including: (1) a relatively large sample of exclusively middle-aged individuals (median age 48.0 years, IQR: 44.5, 54.0), which is important for gaining an insight into the preclinical stage of dementia [20], (2) use of validated and standardized cognitive tests including the PAL test, which is proven to be a sensitive test for cognitive decline [38, 39]. More importantly, this test was found be superior in detecting cognitive decline than the Mini-Mental State Examination (MMSE) [40], the most widely used cognitive screening tool in both clinical settings and epidemiological studies, and (4) use of multivariable linear regression analyses adjusted for many potential confounders. However, our study had some limitations including: (1) our study is cross-sectional, which does not enable conclusions about the causal relationship between sUA and cognitive function, (2) we could not investigate the association between UA and other cognitive function domains (due to unavailability of the data), (3) our study is based on a Qatari database (Qatari Biobank) which does not enable generalization of the findings, and (4) some confounds such as diet and medicine could not be completely eliminated.

## Conclusions

Our current findings of negative association between sUA and cognition support those of previous studies in elderly (> 60 years), and extend the evidence for such association to a younger population (< 60 years old). The findings provide insight into potential prevention and therapy of the clinical dementia. However, prospective studies on middle-aged participants in other regions are needed to verify the generalizability of the findings and determine the causal relations between UA and cognition. Such studies should assess as many cognitive domains as possible and adjust for potential demographic and health confounders for better understanding of the association between UA and cognitive function. 

## Supplementary Information

Below is the link to the electronic supplementary material.

Supplementary file1 (DOCX 40 kb)

Supplementary file2 (DOCX 41 kb)

Supplementary file3 (DOCX 33 kb)

## Data Availability

The data of this study will be available whenever required.
