# Supplementary material for: Higher serum uric acid is associated with poorer cognitive performance in healthy middle-aged people: a cross-sectional study
Source: Intern Emerg Med. 2023 Jun 17;18(6):1701–9. doi: 10.1007/s11739-023-03337-1 (PMC10504193; doi:10.1007/s11739-023-03337-1)
Supplement: Supplementary file 1 — Supplementary file1 (DOCX 40 kb) [file 11739_2023_3337_MOESM1_ESM.docx]

**Supplementary Table 1 – characteristics of female participants**

| Factor | Level | Normal UA | High UA | p-value |
| --- | --- | --- | --- | --- |
| N |  | 463 | 25 |  |
| Age, mean (SD) |  | 49.0 (5.3) | 52.0 (6.2) | 0.007 |
| Age category | 40-49years | 267 (57.7%) | 10 (40.0%) | 0.020 |
|  | 50-59years | 183 (39.5%) | 12 (48.0%) |  |
|  | 60-69years | 13 (2.8%) | 3 (12.0%) |  |
| Nationality | Non-Qatari | 39 (8.4%) | 2 (8.0%) | 0.94 |
|  | Qatari | 424 (91.6%) | 23 (92.0%) |  |
| Education | Primary or below | 54 (11.7%) | 2 (8.0%) | 0.77 |
|  | Secondary | 125 (27.0%) | 6 (24.0%) |  |
|  | Tertiary | 284 (61.3%) | 17 (68.0%) |  |
| BMI, mean (SD) |  | 31.0 (5.5) | 33.3 (5.5) | 0.041 |
| Smoking | Non-smoker | 153 (96.8%) | 10 (100.0%) | 0.57 |
|  | Current smoker | 5 (3.2%) | 0 (0.0%) |  |
| shisha | No | 156 (95.1%) | 10 (100.0%) | 0.47 |
|  | Yes | 8 (4.9%) | 0 (0.0%) |  |
| Diabetes | No | 350 (75.6%) | 19 (76.0%) | 0.96 |
|  | Yes | 113 (24.4%) | 6 (24.0%) |  |
| Hypertension | No | 415 (89.6%) | 24 (96.0%) | 0.30 |
|  | Yes | 48 (10.4%) | 1 (4.0%) |  |
| Memory performance score, mean (SD) |  | 48.9 (28.9) | 34.5 (19.1) | 0.014 |
| Memory performance score, median (IQR) |  | 48.6 (23.5, 75.5) | 31.2 (25.8, 46.2) | 0.018 |
| Reaction performance score, mean (SD) |  | 45.2 (27.7) | 57.0 (23.8) | 0.038 |
| Reaction performance score, median (IQR) |  | 44.9 (19.6, 69.5) | 64.9 (35.1, 78.4) | 0.031 |
