# Supplementary material for: Higher serum uric acid is associated with poorer cognitive performance in healthy middle-aged people: a cross-sectional study
Source: Intern Emerg Med. 2023 Jun 17;18(6):1701–9. doi: 10.1007/s11739-023-03337-1 (PMC10504193; doi:10.1007/s11739-023-03337-1)
Supplement: Supplementary file 2 — Supplementary file2 (DOCX 41 kb) [file 11739_2023_3337_MOESM2_ESM.docx]

**Supplementary Table 2 – characteristics of male participants**

| Factor | Level | Normal UA | High UA | p-value |
| --- | --- | --- | --- | --- |
| N |  | 282 | 161 |  |
| Age, mean (SD) |  | 48.5 (5.5) | 47.4 (4.9) | 0.032 |
| Age category | 40-49years | 167 (59.2%) | 116 (72.0%) | 0.024 |
|  | 50-59years | 109 (38.7%) | 42 (26.1%) |  |
|  | 60-69years | 6 (2.1%) | 3 (1.9%) |  |
| Nationality | Non-Qatari | 46 (16.3%) | 20 (12.4%) | 0.27 |
|  | Qatari | 236 (83.7%) | 141 (87.6%) |  |
| Education | Primary or below | 11 (3.9%) | 3 (1.9%) | 0.19 |
|  | Secondary | 89 (31.7%) | 42 (26.1%) |  |
|  | Tertiary | 181 (64.4%) | 116 (72.0%) |  |
| BMI, mean (SD) |  | 27.9 (4.4) | 30.2 (5.1) | <0.001 |
| Smoking | Non-smoker | 182 (69.5%) | 106 (70.7%) | 0.88 |
|  | Current smoker | 77 (29.4%) | 43 (28.7%) |  |
|  | Unknown | 3 (1.1%) | 1 (0.7%) |  |
| shisha | No | 187 (71.9%) | 105 (70.9%) | 0.83 |
|  | Yes | 73 (28.1%) | 43 (29.1%) |  |
| Diabetes | No | 202 (71.6%) | 131 (81.4%) | 0.023 |
|  | Yes | 80 (28.4%) | 30 (18.6%) |  |
| Hypertension | No | 257 (91.1%) | 135 (83.9%) | 0.021 |
|  | Yes | 25 (8.9%) | 26 (16.1%) |  |
| Memory performance score, mean (SD) |  | 56.2 (28.8) | 52.4 (27.3) | 0.17 |
| Memory performance score, median (IQR) |  | 61.3 (32.1, 80.9) | 53.1 (33.1, 73.5) | 0.13 |
| Reaction performance score, mean (SD) |  | 57.7 (28.1) | 56.9 (28.8) | 0.77 |
| Reaction performance score, median (IQR) |  | 57.2 (34.7, 84.3) | 56.6 (33.7, 83.8) | 0.81 |
