# Supplementary material for: Higher serum uric acid is associated with poorer cognitive performance in healthy middle-aged people: a cross-sectional study
Source: Intern Emerg Med. 2023 Jun 17;18(6):1701–9. doi: 10.1007/s11739-023-03337-1 (PMC10504193; doi:10.1007/s11739-023-03337-1)
Supplement: Supplementary file 3 — Supplementary file3 (DOCX 33 kb) [file 11739_2023_3337_MOESM3_ESM.docx]

**Supplementary Table 3- Multivariable linear regressions for the association between uric acid quartiles and episodic memory test and reaction time test**

| 95% Confidence Interval | P-value | Coefficient |  |
| --- | --- | --- | --- |
| Memory test performance | | | |
| Uric acid categories | | | |
| -1.84 to 3.20 | 0.474 | -6.89 | Group 2,  n= 226 |
| 0.96 to 6.37 | 0.729 | -4.46 | Group 3,  n= 233 |
| -4.16 to 1.75 | 0.168 | -10.07 | Group 4,  n= 232 |
| 41.09 to 54.45 | 0.000 | 27.73 | Constant |
| Reaction Test Score | | | |
|  |  |  | Uric acid categories |
| - -124.25 to 156.41 | 0.822 | 16.08 | Group 2,  n= 226 |
| -198.45 to 102.62 | 0.532 | -47.91 | Group 3,  n= 233 |
| -208.09 to 120.81 | 0.603 | -43.64 | Group 4,  n= 232 |
| 330.19 to 1072.94 | <0.001 | 701.57 | Constant |

**Supplementary Table 3:** showing results of two multivariable linear regression models between sUA quartiles and cognitive function adjusted for age, gender, BMI, education, diabetes and hypertension.

Participants were divided among quartiles based on their sUA level, group 1 included participants with sUA < 243 µmol/L, group 2 included participants with sUA < 294 µmol/L, group 3 included participants with sUA < 344 µmol/L, and group 4 included participants with sUA ≥ 344 µmol/L.
